# Nevus of Ota – an intraoral presentation: a case report

**DOI:** 10.1186/s13256-019-2101-0

**Published:** 2019-06-07

**Authors:** Jennifer Maguire, Deborah Holt

**Affiliations:** 1 Our Lady’s Children’s Hospital Crumlin, Dublin, Ireland; 2 Oral Medicine Department, Liverpool University Dental Hospital, Liverpool, UK

**Keywords:** Oral, Pigmentation, Nevus, Ota, Oculodermal, Buccal, Mucosa

## Abstract

**Background:**

Nevus of Ota or “oculodermal melanocytosis” is a rare congenital hamartoma of dermal melanocytes causing a blue-gray hyperpigmentation of the eye and surrounding structures. The condition, originally described by Ota and Tanino in 1939, mainly affects the ophthalmic and maxillary divisions of the trigeminal nerve. We describe the first reported case of unilateral oculodermal melanocytosis in a Caucasian woman with oral buccal mucosal involvement. Oral involvement of nevus of Ota is very rare.

**Case presentation:**

A 48-year-old Caucasian woman was referred by the dermatology division to the oral medicine department at the University of Liverpool School of Dentistry with new-onset oral pigmentation to the left buccal mucosa. The patient had a previous diagnosis of oculodermal nevus.

**Conclusion:**

An incisional biopsy of the left buccal mucosa was completed. The report stated that histological and immunohistochemical features were in keeping with a blue nevus, but within the context of the preexisting occulodermal pigmentation, a diagnosis of oculodermal melanocytosis, also known as “nevus of Ota,” was made. The patient will be kept under review in the oral medicine department because the progression of the lesion on the left buccal mucosa requires active monitoring owing to the potential for malignant change. The patient also requires regular review in the dermatology and ophthalmology divisions.

## Background

Nevus of Ota or “oculodermal melanocytosis” is a rare congenital hamartoma of dermal melanocytes causing a blue-gray hyperpigmentation of the eye and surrounding structures [1]. Originally described by Ota and Tanino in 1939, it mainly affects the ophthalmic and maxillary divisions of the trigeminal nerve and is most prevalent in the Japanese population, with an incidence reported between 0.2% and 1% [2]. Oral involvement of the nevus of Ota is very rare [3]. We describe the first reported case of unilateral oculodermal melanocytosis with oral buccal mucosal involvement in a Caucasian woman. It is important to be aware that nevus of Ota can present orally.

## Case presentation

A 48-year-old Caucasian woman was referred to the oral medicine department at the University of Liverpool School of Dentistry with new-onset oral pigmentation to the left buccal mucosa. Her past medical history revealed a diagnosis of “oculodermal nevus.” She recalled having pigmentation in her left eye from birth and pigmentation of skin of the left face since the age of 13 years, for which she received laser treatment for cosmetic purposes. The patient also reported annual monitoring of a benign intracranial tumor along with close monitoring by ophthalmology and dermatology divisions. She did not take any regular medications. She did not smoke or consume alcohol. She is single and lives on her own with no dependents. She lives close to her mother, who attended the appointments with her.

**Fig. 1:**
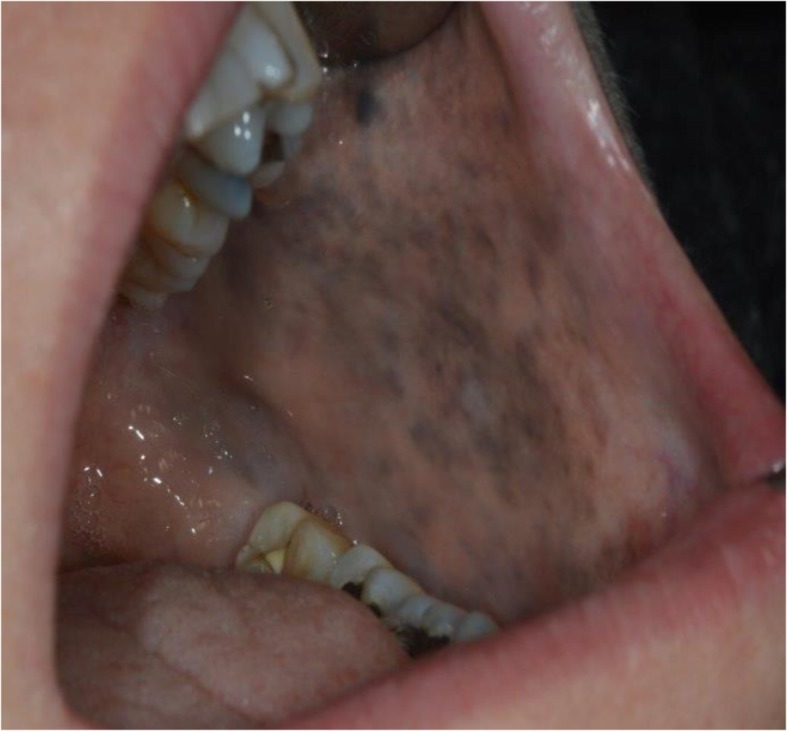
Naevus of Ota affecting the left buccal mucosa

**Fig. 2:**
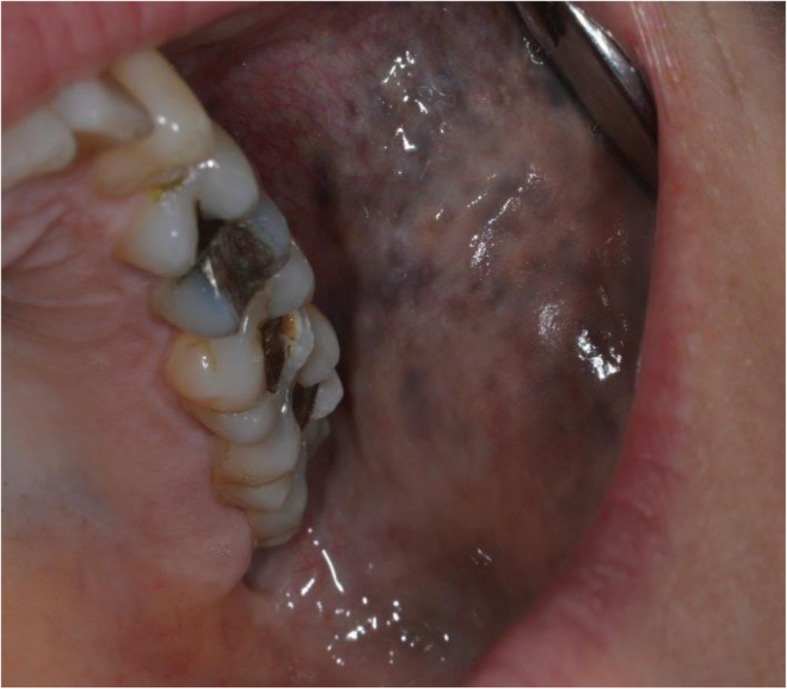
Naevus of Ota affecting the left buccal mucosa and palate

**Fig. 3:**
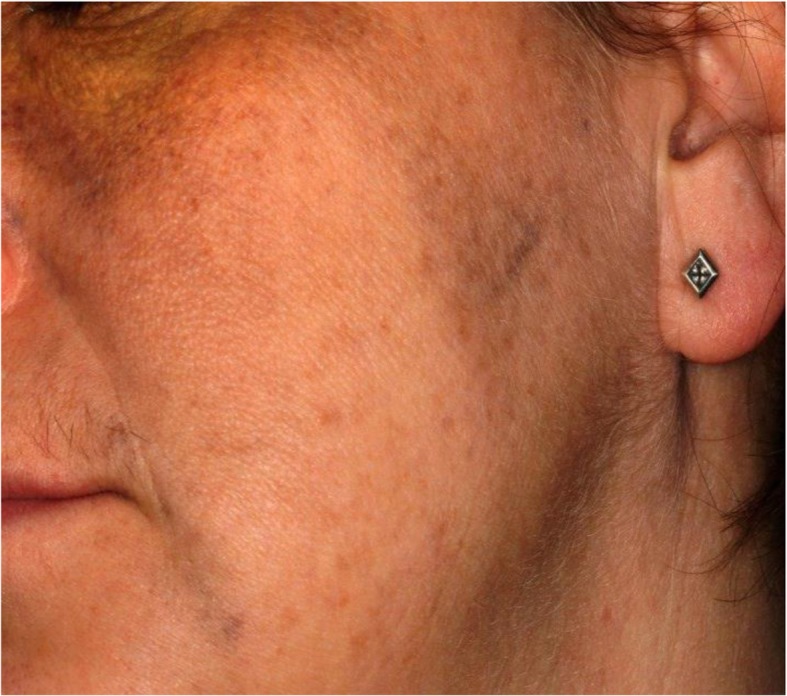
Naevus of Ota affecting the left Infra-Orbital and Zygomatic region

**Fig. 4:**
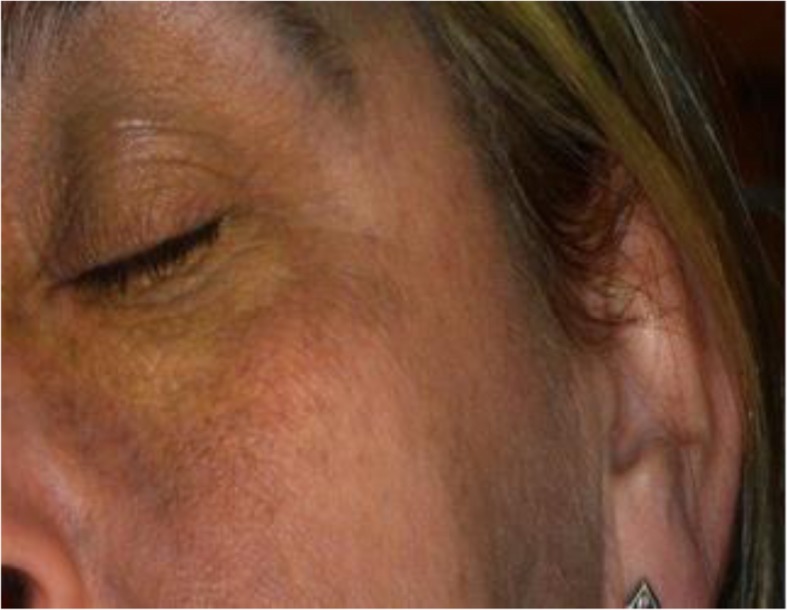
Post laser scarring in the left Infra-Orbital region

**Fig. 5:**
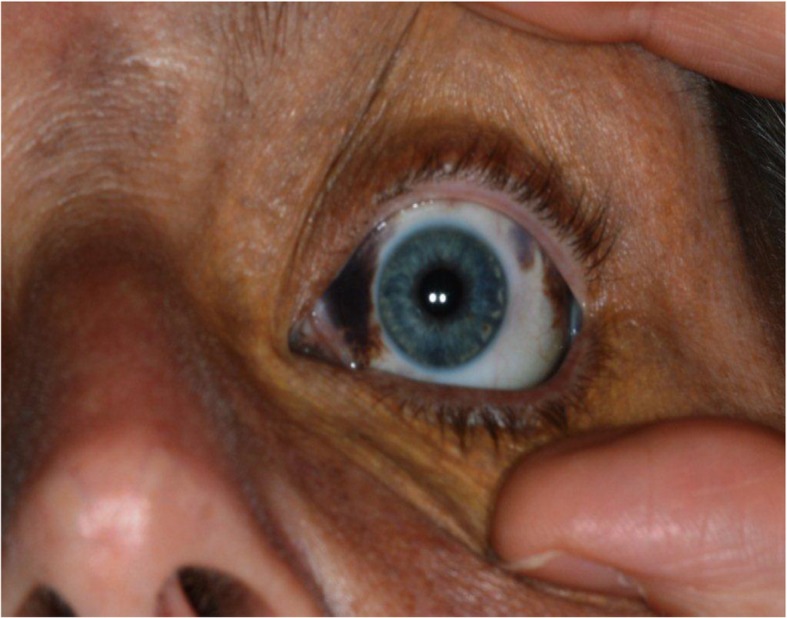
Naevus of Ota afffecting the sclera of the left eye and post laser scarring

On examination, a subtle but diffusely speckled bluish pigmentation was observed to the left midface involving the infraorbital and zygomatic regions. A post–laser therapy yellow hue was noted on the left periorbital skin. Pigmentation of the sclera and conjunctiva was also observed. Intraorally, an inhomogeneous, blue-gray, diffuse hyperpigmentation affecting the entire left buccal mucosa was noted. Mild pigmentation of the left hard palate was also noted (Figs. 1, 2, 3, 4 and 5).

An incisional biopsy of the left buccal mucosa was completed. The report stated that histological and immunohistochemical features were in keeping with a blue nevus, but within the context of the preexisting occulodermal pigmentation, a diagnosis of oculodermal melanocytosis, also known as “nevus of Ota,” was made. No other investigations were required. The patient will be kept under 6-monthly review with the oral medicine department because the progression of the lesion on the left buccal mucosa requires active monitoring owing to the potential for malignant change.

## Discussion and conclusions

Nevus of Ota represents a unilateral dermal melanosis in the distribution of the trigeminal nerve, where it is usually confined to the ophthalmic and maxillary divisions. The forehead, temple, periorbital area, cheek, and nose are most commonly involved. Melanin pigmentation involves the eye in about 50% of cases [4]. Rarely is the pigmentation bilateral with large areas of the face and oral mucous membranes being affected [2].

A literature search of PubMed and Embase revealed only two cases (in India) of nevus of Ota affecting the buccal mucosa [5, 6]. Intraoral presentations more frequently involve the palatal mucosa.

The prevalence of this condition is greatest in the Asian continent, affecting up to 1% of the Japanese population [2]. One study has reported an incidence of 0.038% in Caucasians, although this is poorly documented [7]. About 85% cases of nevus of Ota occur in females.

Use of lasers for treatment of nevus of Ota has become helpful in the management of dermal nevi. Currently, Q-switched lasers are the most studied, and they have demonstrated positive results for nevus of Ota [8]. Lasers are more effective in light-skinned individuals; however, recurrence can be more common and may result in a darker hue.

Our patient had received laser therapy 20 years earlier and had a good outcome. She has also been referred to the cosmetic camouflage clinic. The patient has seen a makeup artist and uses cosmetics to cover the pigmentation. Long-term follow-up, especially in the ophthalmology division (6-monthly) [9] and neurosurgery division, is required because of the risk of ocular melanoma and central nervous system neoplasia, although this is rare. The patient’s intracranial lesion is unrelated to the nevus of Ota. There is also an increased risk of glaucoma associated with nevus of Ota in 10% of patients [10]. A number of cases of malignant melanoma are also reported in the literature, and careful observation is necessary, particularly in Caucasians, in whom malignant degeneration seems to occur with a disproportionate frequency [11]. It is recommended that these patients be reviewed annually by a dermatologist. It is also of note that there are possible molecular explanations for the risk of malignancy, including mutations of *GNAQ* and *BAP1* genes [12]. This will require further research.
