# Correction to: Nevus of Ota – an intraoral presentation: a case report

**DOI:** 10.1186/s13256-019-2194-5

**Published:** 2019-08-07

**Authors:** Jennifer Maguire, Deborah Holt

**Affiliations:** 1 Our Lady’s Children’s Hospital Crumlin, Dublin, Ireland; 2 Oral Medicine Department, Liverpool University Dental Hospital, Liverpool, UK

**Correction to: Med Case Reports**

**Fig. 1:**
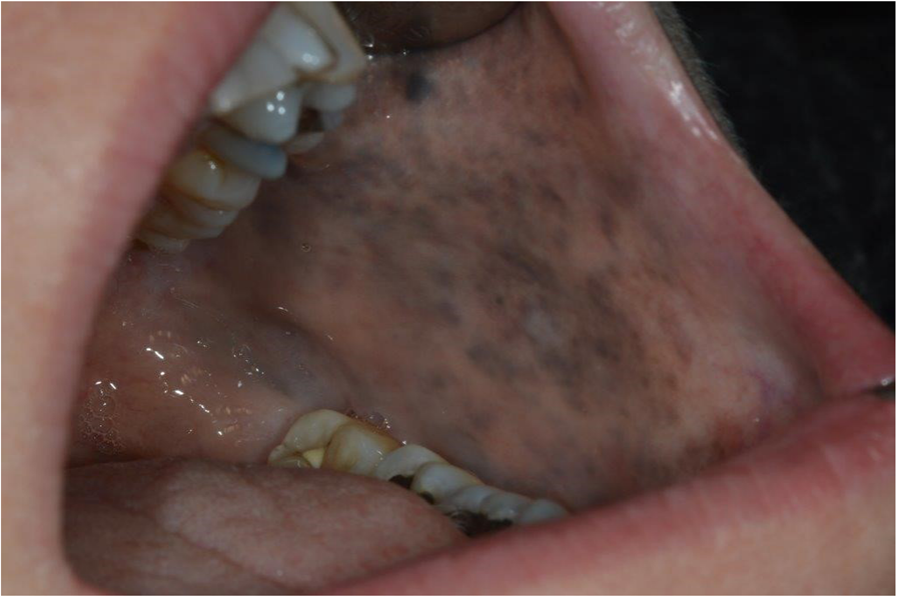
Naevus of Ota affecting the left buccal mucosa

**Fig. 2:**
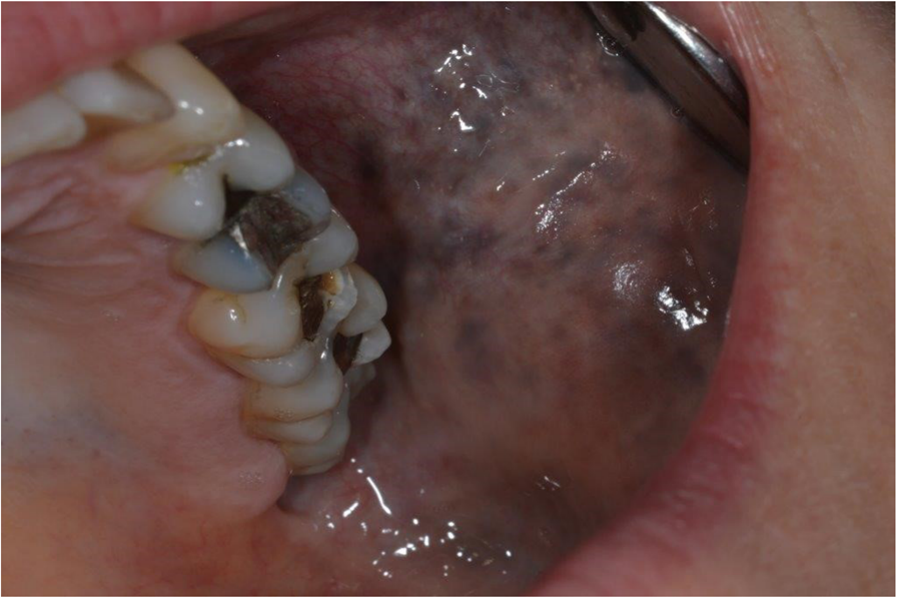
Naevus of Ota affecting the left buccal mucosa and palate

**Fig. 3:**
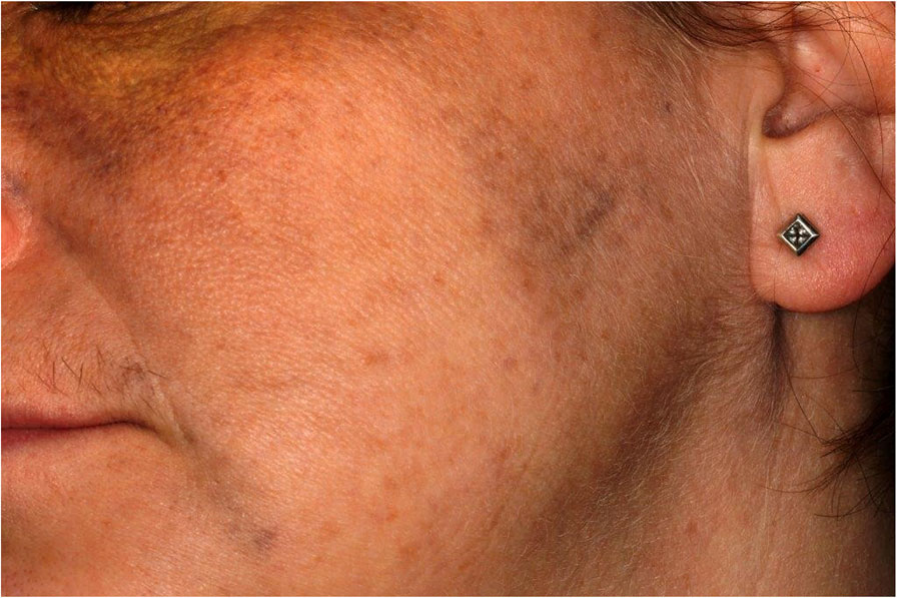
Naevus of Ota affecting the left Infra-Orbital and Zygomatic region

**Fig. 4:**
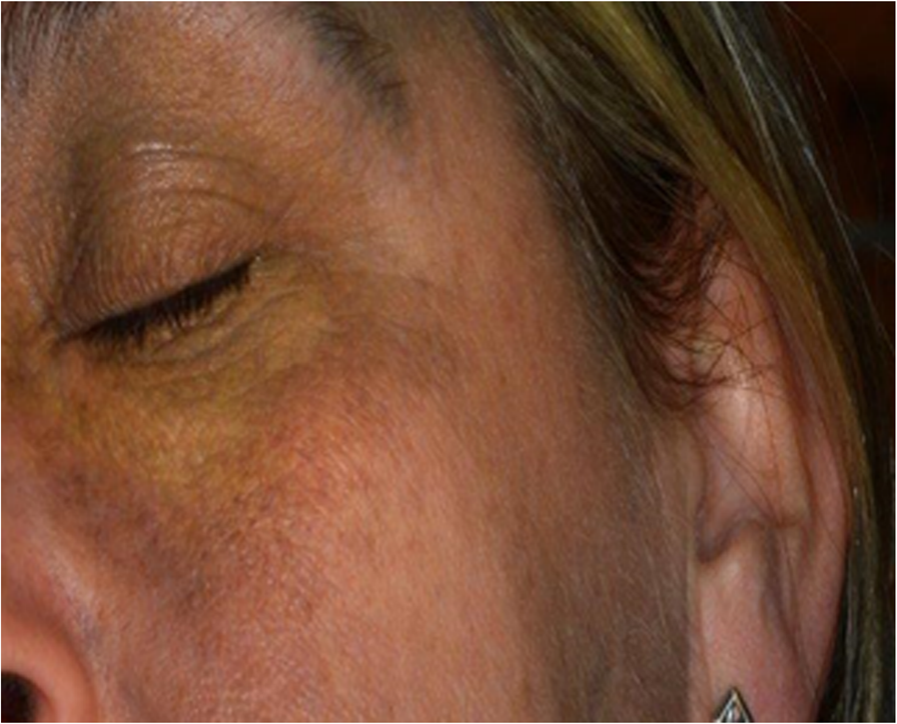
Post laser scarring in the left Infra-Orbital region

**Fig. 5:**
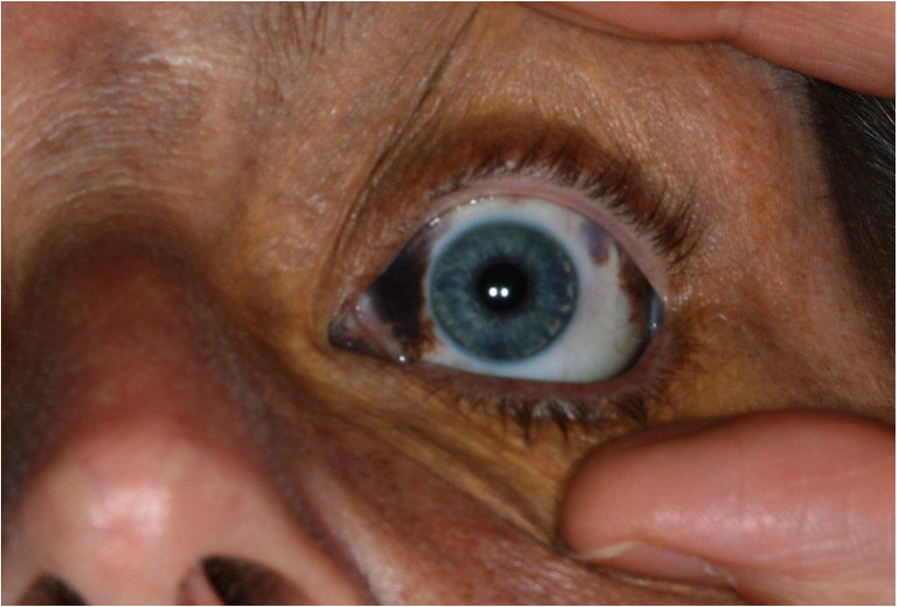
Naevus of Ota afffecting the sclera of the left eye and post laser scarring

**https://doi.org/10.1186/s13256-019-2101-0**

In the publication of this article [1], the figures were accidentally omitted due to an error. This has now been updated in the original article within the Case presentation section.
